# Supplementary material for: NOC1 is a direct MYC target, and its protein interactome dissects its activity in controlling nucleolar function
Source: Front Cell Dev Biol. 2023 Dec 28;11:1293420. doi: 10.3389/fcell.2023.1293420 (PMC10782387; doi:10.3389/fcell.2023.1293420)
Supplement: Supplementary file 2 [file Table1.DOCX]

Supplementary Figure 1


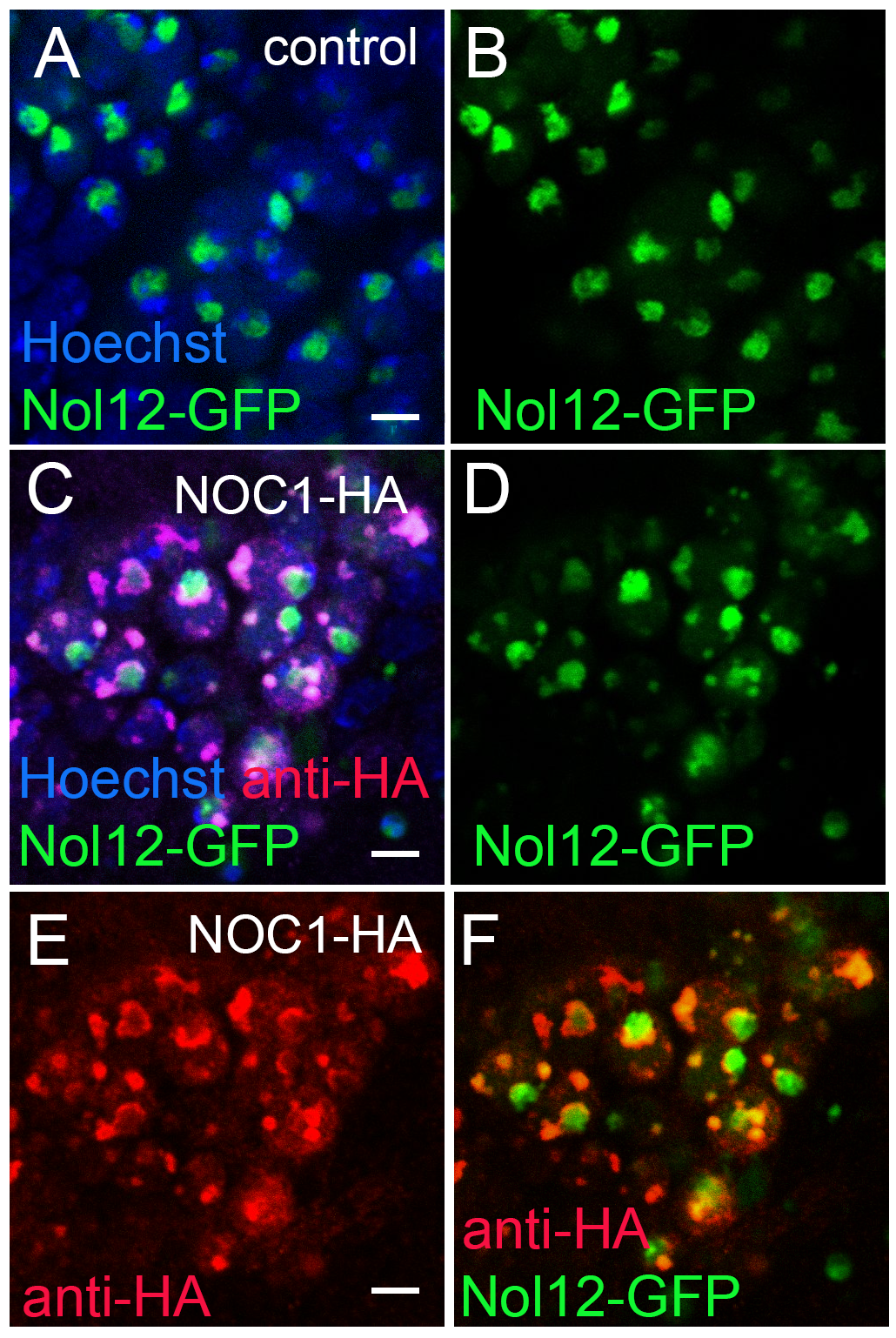


**Expression of NOC1 colocalizes with Nol12 in the nucleolus**. Confocal images of cells from the wing imaginal discs of third instar larvae expressing Nol12 as *UAS-Nol12-GFP* fusion protein using the *rotund* promoter *rn-Gal4.* In A-B, Nol12-GFP localization in the nucleolus of cells from control *w^1118^* animals. In C-F Nol12-GFP expression is shown together with NOC1-HA (red) visualized using an ant-HA antibody. Nuclei are stained using Hoechst and visualized in blue. Scale bars represent 5 μm.
